# The impact of drug use patterns on mortality among polysubstance users in a Canadian setting: a prospective cohort study

**DOI:** 10.1186/1471-2458-14-1153

**Published:** 2014-11-06

**Authors:** Anna Hayden, Kanna Hayashi, Huiru Dong, Michael-John Milloy, Thomas Kerr, Julio SG Montaner, Evan Wood

**Affiliations:** British Columbia Centre for Excellence in HIV/AIDS, St. Paul’s Hospital, 608 - 1081 Burrard Street, Vancouver, BC V6Z 1Y6 Canada; Department of Medicine, University of British Columbia, 317-2194 Health Sciences Mall, Vancouver, BC V6T 1Z3 Canada

**Keywords:** Mortality, Injection drug use, Cocaine, Vancouver, Cohort study

## Abstract

**Background:**

Illicit drug use is a well-established risk factor for morbidity and mortality. However, few studies have examined the impact of different drug use patterns on mortality among polysubstance using populations. This study aimed to identify drug-specific patterns of mortality among a cohort of polysubstance using persons who inject drugs (PWIDs).

**Methods:**

PWIDs in Vancouver, Canada were prospectively followed between May 1996 and December 2011. Participants were linked to the provincial vital statistics database to ascertain mortality rates and causes of death. We used multivariate Cox proportional hazards regression to investigate the relationships between drug use patterns (daily alcohol use, heroin injection and non-injection use, cocaine injection, amphetamine injection and non-injection use, crack smoking and speedball injecting) and time to all-cause mortality.

**Results:**

2330 individuals were followed for a median of 61 months (inter-quartile range: 33 – 112). In total, 466 (19.1%) individuals died for an incidence density of 3.1 (95% confidence interval [CI]: 2.8 – 3.4) deaths per 100 person-years. In multivariate analyses, after adjusting for HIV infection and other potential confounders, only daily cocaine injection remained independently associated with all-cause mortality (adjusted hazard ratio [AHR] = 1.36, 95% CI: 1.06 – 1.76).

**Conclusions:**

Although heroin injecting is traditionally viewed as carrying the highest risk of mortality, in this setting, only daily cocaine injecting was associated with all-cause mortality. These findings highlight the urgent need to identify novel treatments and harm reduction strategies for cocaine injectors.

## Background

Illicit drug use has been well established as a risk factor for adverse health outcomes including premature mortality [1–6]. Previous studies have indicated that fatal overdose accounts for a majority of deaths in illicit drug-using populations, particularly among persons who inject drugs (PWIDs) [3–5, 7, 8]. Much of the existing literature has highlighted opioid use associated with fatal overdose [1, 7, 9, 10]. One meta-analysis found that illicit opiate users have a mortality rate thirteen times that of the general population [1]. An increased mortality rate has also been demonstrated among users of other substances, including cocaine and amphetamines [4, 6, 11, 12].

Earlier studies of mortality among PWIDs have often been limited by the fact that they were restricted to cross-sectional examinations of toxicology reports among deceased individuals where important potential confounders, such as human immunodeficiency virus (HIV) infection or other socio-demographic comorbidities, like homelessness, were difficult to control for [2, 7, 13, 14]. While longstanding prospective cohort studies of PWIDs exist in a number of regions, some of which have adjusted for potential confounders, many have been undertaken in areas where a limited number of specific drugs are primarily used (e.g. primarily heroin or cocaine users) [7, 10, 11, 15–17].

The Downtown Eastside of Vancouver (DTES), Canada is well known for its concentration of poly-substance use, high prevalence of hepatitis C virus (HCV) and HIV infection and high rates of homelessness [18, 19]. The DTES is among the most impoverished neighborhoods in Canada with an estimated 5000 PWIDs residing in the area [18, 19]. Various patterns of drug use in this population have been described in previous studies including high rates of cocaine injecting [20], crack cocaine smoking [21], amphetamine injecting [22] and heroin injecting [23]. Accordingly, it provides an excellent environment to examine the contribution of specific drug use patterns on mortality. Therefore, the present study aimed to identify drug specific patterns of mortality among a cohort of polysubstance using PWIDs in Vancouver.

## Methods

The Vancouver Injection Drug Users Study (VIDUS) and AIDS Care Cohort to Evaluate Access to Survival Services (ACCESS) are open prospective cohorts of drug users in Vancouver. The recruitment and follow-up procedures for the two studies are identical to allow for analyses of merged data, with the only differences being that HIV-positive individuals are followed in ACCESS whereas HIV-negative individuals are followed in VIDUS and that ACCESS includes non-injecting drug users. In both studies the primary modes of enrollment were self-referral, word of mouth, and street outreach. Detailed sampling and recruitment procedures for these two cohorts have been described elsewhere [20, 24, 25].

To be eligible, participants were 18 years of age or older, had used illicit drugs other than cannabinoids in the previous month and resided in the greater Vancouver region. All participants provided written informed consent. Participants were given a stipend ($20 CDN) at each study visit for their time and transportation. The study was approved by the University of British Columbia/Providence Healthcare Research Ethics Board.

At baseline and semianually thereafter, participants completed an interviewer-administered questionnaire that elicited a range of data, including demographic characteristics, injection and non-injection drug use, and sexual risk behaviors. In addition, venous blood samples were drawn at each visit and tested for HIV and HCV antibodies. All participants had private interviews and were offered both pre- and post-test counseling with trained nurses. Referral for free healthcare was provided to those who tested HIV positive and these individuals were subsequently followed in ACCESS.

We ascertained all-cause mortality rates and underlying causes of death among participants through a confidential record linkage with the British Columbia Vital Statistics Agency and through ongoing follow-up with contacts provided by participants. The Vital Statistics database recorded causes of death according to the International Classification of Diseases (ICD), 10th edition.

The present study included PWIDs who were recruited and completed at least one follow up visit between May 1996 and December 2011. To avoid potential bias relating to long durations between the last study visit where behavioural information was assessed and the date of death (i.e. loss to regular follow up), individuals who were identified as deceased more than 12 months after the the last follow up visit were censored on the date of the last follow up.

The primary endpoint in this analysis was all-cause mortality. The primary explanatory variables of interest included a number of substance use behaviors in the previous six months, including at least daily alcohol use, at least daily cocaine injection, at least daily heroin injection and non-injection use, at least daily amphetamine injection and non-injection use, at least daily crack cocaine smoking and at least daily speedball injecting (a mixture of cocaine and heroin). Additionally, we examined risk associated with non-daily heroin and amphetamine use defined as any report of use in the last six months. Potential confounders that were considered included: age (per 10 years older); gender (male vs. female), time since first injection (per 10 years longer); ethnicity (Caucasian vs. others); HIV serostatus (positive vs. negative); and unstable housing in the previous six months. As previously, unstable housing was defined as living in one of the DTES’ single room occupancy hotels, shelters or other transitional housing, or living on the street [26, 27].

As an initial analysis, we used the Chi-square test and Wilcoxon rank sum test to compare the baseline characteristics of the included and excluded individuals, and those who did and did not report daily cocaine injection in the previous six months among the included sample based on an interest in the role of cocaine injecting on mortality. All-cause mortality rate and 95% confidence interval [CI] were calculated using the Poisson distribution.

Next, we used bivariate and multivariate Cox proportional hazards regression to examine the relationship between substance use patterns and time to death. All behavioral variables including substance use patterns, unstable housing and HIV serostatus were treated as time-varying variables. The multivariate model was fit using an *a priori* defined protocol whereby all illicit drug use variables were entered into the multivariate model. Variables found to be significantly associated with time to all-cause mortality in bivariate analyses at *p* <0.10 were entered into the multivariate model as potential confounders.

We also conducted a sub-analysis where we restricted the dependent variable to accidental poisonings (ICD-10: X40–44) and other accidental causes of death to specifically examine if drug use patterns were associated with accidental death. All statistical analyses were performed using SAS software version 9.3 (SAS, Cary, NC). All *p*-values were two-sided.

## Results

A total of 2597 individuals were recruited between May 1996 and December 2011, among whom 267 were excluded as a result of no follow-up information. In comparing the study sample to those that were excluded, the excluded sample was younger, less likely to be HIV positive and was less likely to inject cocaine and speed ball (all *p <*0.05).

The remaining 2330 (89.7%) were followed for a median of 61 months (inter-quartile range [IQR]: 33 – 112). Table 1 shows the baseline characteristics of the study sample. As shown, at baseline, 1550 (66.5%) were men, 640 (27.5%) were HIV positive, and 1424 (61.1%) were Caucasian. The median age was 37.3 years (IQR: 29.4 – 43.7), and the median time since first injection was 14.4 years (IQR: 6.2 – 24.2). At baseline, 550 (23.6%) of the study sample consumed alcohol daily in the previous six months, 901 (38.7%) injected heroin daily, 97 (4.2%) smoked heroin daily, 1588 (68.2%) injected cocaine daily, 51 (2.2%) injected amphetamines daily, 7 (0.3%) smoked amphetamines daily, 560 (24.0%) smoked crack cocaine daily, and. 288 (12.4%) injected speedball daily.

**Table 1:** **Baseline demographics of the study population stratified by daily cocaine injection in the past 6 months (n = 2330)**

Characteristic	Total (%)	Daily cocaine injection			
		Yes (%)	No (%)	Odds ratio (95% CI)	***p***value
	(***n***= 2330)	(***n***= 1588)	(***n***= 728)		
**HIV serostatus**					
Positive	640 (27.5)	212 (29.1)	426 (26.8)	1.12 (0.92 – 1.36)	0.247
Negative	1688 (72.5)	515 (70.7)	1161 (73.1)		
**Gender**					
Male	1550 (66.5)	444 (61.0)	1098 (69.1)	0.70 (0.58 – 0.84)	<0.001
Female	780 (33.5)	284 (39.0)	490 (30.9)		
**Ethnicity**					
Caucasian	1424 (61.1)	413 (56.7)	1001 (63.0)	0.77 (0.64 – 0.92)	0.004
Other	906 (38.9)	315 (43.3)	587 (37.0)		
Unstable housing*					
Yes	1637 (70.3)	556 (76.4)	1072 (67.5)	1.62 (1.32 – 1.99)	<0.001
No	677 (29.1)	163 (22.4)	509 (32.1)		
**Median age (IQR)**					
Per 10 year older	37.3 (14.3)	35.3 (13.4)	38.4 (14.1)	0.76 (0.69 – 0.83)	<0.001
**Median time since first injection (IQR)**					
Per 10 year longer	14.4 (18.0)	14.1 (16.3)	14.6 (18.8)	0.91 (0.84 – 0.99)	0.033
Daily alcohol use*					
Yes	550 (23.6)	179 (24.6)	368 (23.2)	1.10 (0.88 – 1.33)	0.450
No	1775 (76.2)	547 (75.1)	1217 (76.6)		
Daily heroin injection*					
Yes	901 (38.7)	345 (47.4)	555 (35.0)	1.68 (1.40 – 2.00)	<0.001
No	1424 (61.1)	383 (52.6)	1033 (65.1)		
Daily heroin (non-injection)*					
Yes	97 (4.2)	30 (4.1)	66 (4.2)	0.99 (0.64 – 1.54)	0.969
No	2230 (95.7)	697 (95.7)	1520 (95.7)		
Daily amphetamine injection*					
Yes	51 (2.2)	7 (1.0)	44 (2.8)	0.34 (0.15 – 0.76)	0.006
No	2274 (97.6)	721 (99.0)	1542 (97.1)		
Daily amphetamine (non-injection)*					
Yes	7 (0.3)	1 (0.1)	6 (0.4)	0.36 (0.04 – 3.02)	0.445^†^
	2322 (99.7)	727 (99.9)	1581 (99.6)		
Daily crack cocaine smoking*					
Yes	560 (24.0)	137 (18.8)	420 (26.5)	0.65 (0.52 – 0.80)	<0.001
No	1767 (75.8)	589 (80.9)	1167 (73.5)		
Daily speedball injection*					
Yes	288 (12.4)	216 (29.7)	71 (4.5)	9.01 (6.77 – 12.00)	<0.001
No	2038 (87.5)	512 (70.3)	1516 (95.5)		

*Refers to activities in the six months prior to interview. IQR = Interquartile range. ^†^Fisher’s Exact Test.

Compared to non-daily cocaine injectors, those who reported daily cocaine injection at baseline were more likely to be younger (odds ratio [OR] = 0.76, 95% CI: 0.69 – 0.83), to reside in unstable housing (OR = 1.62, 95% CI: 1.32 – 1.99), and to have less time since first injection (OR = 0.91, 95% CI: 0.84 – 0.99). They were less likely to be male (OR = 0.70, 95% CI: 0.58 – 0.84) and report Caucasian ethnicity (OR = 0.77, 95% CI: 0.64 – 0.92). In terms of drug use patterns, daily cocaine injection was significantly and positively associated with daily heroin injection (OR = 1.68, 95% CI: 1.40 – 2.00) and speedball injection (OR = 9.01, 95% CI 6.77 – 12.00), and negatively associated with daily amphetamine injection (OR = 0.34, 95% CI: 0.15 – 0.76), and daily crack smoking (OR = 0.65, 95% CI: 0.52 – 0.80).

In total, 466 (19.1%) individuals died for an incidence density of 3.1 (95% CI: 2.8 – 3.4) deaths per 100 person-years. The primary underlying causes of death included accidental poisonings (22.6%) and HIV disease (18.5%). The remaining 100 classified categories including assault (2.3%) and lung malignancy (2.4%) are varied and each individually contributed less than 5%, with most contributing less than 1% to all-cause mortality.

Table 2 shows results of the bivariate and multivariate Cox regression analyses of all-cause mortality. In the bivariate analysis, daily cocaine injection was significantly and positively associated with time to all-cause mortality with a hazard ratio (HR) of 1.41 (95% CI: 1.12 – 1.78) whereas daily heroin injection was significantly and negatively associated with the ouctome with a HR of 0.75 (95% CI: 0.60 – 0.95). Daily alcohol use (HR = 0.98, 95% CI: 0.77 – 1.25), daily heroin non-injection use (HR = 1.12, 95% CI: 0.51 – 2.44), amphetamine injection (HR = 0.39, 95% CI: 0.10 – 1.54) and non-injection use (HR = 1.13, 95% CI: 0.29 – 4.50), daily crack cocaine smoking (HR = 0.91, 95% CI: 0.74 – 1.12) and daily speedball injection (HR = 1.02, 95% CI: 0.69 – 1.52) were not significantly associated with time to all-cause mortality. A bivariate analysis of non-daily heroin use (HR = 0.99, 95% CI: 0.72 -1.37) and amphetamine (HR = 0.47, 95% CI: 0.23 – 1.02) use, which was not included in the tables showed no significant association with mortality.

**Table 2:** **Univariate and multivariate Cox proportional hazard regression analyses of the time to all-cause death among people who inject drugs in Vancouver, Canada (n = 2330)**

	Unadjusted hazard ratio (HR)			Adjusted ^†^ hazard ratio (AHR)		
Variable	HR	(95% CI)	***p***-value	AHR	(95% CI)	***p***-value
Daily alcohol use*	0.98	0.77 – 1.25	0.854	1.00	0.78 – 1.28	0.979
Daily heroin injection*	0.75	0.60 – 0.95	0.018	0.92	0.71 – 1.19	0.502
Daily heroin (non-injection)*	1.12	0.51 – 2.44	0.779	1.44	0.68 – 3.07	0.340
Daily cocaine injection*	1.41	1.12 – 1.78	0.003	1.36	1.06 – 1.76	0.017
Daily amphetamine injection*	0.39	0.10 – 1.54	0.177	0.41	0.10 – 1.72	0.224
Daily amphetamine (non-injection)*	1.13	0.29 – 4.50	0.859	0.94	0.13 – 6.88	0.949
Daily crack cocaine smoking*	0.91	0.74 – 1.12	0.358	0.83	0.67 – 1.04	0.099
**Daily speedball injectionn**	1.02	0.69 – 1.52	0.917	0.98	0.62 – 1.56	0.944

*Refers to activities in the six months prior to interview. ^†^Model was adjusted for HIV serostatus, age, and unstable housing.

In the multivariate analysis, after adjustment for potential confounders including HIV serostatus, age and unstable housing, daily cocaine injection remained independently and positively associated with time to all-cause death (adjusted hazard ratio [AHR] = 1.36, 95% CI: 1.06 – 1.76) whereas the remaining substance use variables were not significantly associated with the outcome.

The sub-analysis whereby the dependent variable was restricted to accidental mortality showed that none of the substance use variables was significantly associated with accidental mortality. The only variable where there was a trend towards an association with accidental mortality was daily crack cocaine injecting, though this was not statistically significant (AHR = 1.36, 95% CI: 0.91– 2.10). Full data are available from the corresponding author.

## Discussion

In the present study, we found that daily cocaine injection was independently associated with all-cause mortality among PWIDs in Vancouver, after adjusting for potential confounders including age, unstable housing and HIV serostatus. These variables are known risk factors for mortality and were included in the analysis in order to adjust for their potential confounding effects [8]. The most common causes of death included accidental poisonings and HIV disease. We did not observe an independent mortality risk associated with daily alcohol use, daily heroin injection or non-injection use, daily amphetamine injection or non-injection use, daily crack cocaine smoking and daily speedball injecting.

Our findings that daily cocaine injection was the only drug use behaviour independently associated with mortality were somewhat unique in the context of previous literature indicating a significant mortality risk associated with heroin injection most commonly due to fatal overdose [5]. Heroin use has been shown to carry the highest mortality risk amongst illicit drugs in some studies [14, 28, 29]. Particularly, non-daily heroin use is thought to carry mortality risk due to diminished tolerance to respiratory depression effects with sporadic use leading to increased of accidental overdose [30]. However, our analysis showed no significant increase in mortality risk in both daily and non-daily heroin users. While the underlying reasons for the discrepancy between these previous studies and our study findings are not entirely clear, a contributing factor may be related to frequency of drug injecting, which was not accounted for in the previous studies [4, 8]. Due to the short half life of cocaine, injectors will often use greater than 20 times in a day, predisposing them to increased risk of infection/bacteremia and other associated negative health effects, while local heroin injectors typically inject only 2 to 4 times per day [20]. Rates of injecting may increase risk of bacterial infection, needle sharing and other potential risks of mortality (e.g. air embolus, cellulitis, etc.) [12, 20, 28].

We also found a differential risk of mortality between cocaine injectors and crack smokers. Some cohort studies have demonstrated an independent mortality risk associated with cocaine use [4, 12] in addition to significant morbidity associated with the use of this substance, including cardiovascular, psychiatric, neurologic disorders and unintentional injuries [16]. Much of the mortality data on cocaine use however does not distinguish between injecting and smoking crack cocaine [16, 17, 28]. Furthermore, the majority of these studies present data in standardized mortality ratios, which allow for a comparison with the general population but not between sub-groups of cocaine-using populations, as performed in the present study. Though both cocaine injection and crack smoking have been shown to be associated with HIV infection in this setting [20, 31], our multivariate analyses suggest that daily cocaine injectors appear to be at an elevated risk of mortality due to the risks associated with injection practices other than HIV infection.

Harm reduction strategies, including a supervised injecting facility, in Vancouver’s DTES have been shown to be successful at attracting cocaine injectors, reducing rates of fatal overdose [32, 33] and reducing syringe sharing and HIV risk behavior [34, 35] among the local PWID population. It is noteworthy that both cocaine and heroin injectors utilize this unique program, [35] which is widely accessible for extended hours. It also connects PWIDs to addiction services, contributing to quicker entry into detoxification programs [36] and leading to increased likelihood of stopping drug injecting [37].

The findings of this study highlight a need to further identify addiction treatment and public health strategies tailored for cocaine injectors. Currently, there is no standard pharmacotherapy proven effective for cocaine addiction, though multiple therapeutic agents, including anticonvulsants and stimulants have been investigated as potential treatments [38, 39]. A recent comprehensive review of human clinical trials for potential novel therapies for treatment of cocaine dependence outlined burgeoning research in this field. It identified multiple promising pharmocotherapies including dopamine agonists, serotonergic agents and GABA-ergic medications and a cocaine vaccine. One promising randomized controlled trial from 2013 demonstrated that topiramate was more efficacious than placebo at reducing weekly cocaine use [40]. Additionally, contingency management strategies have been presented as possible interventions. One such study showed that employment-based abstinence reinforcement could lead to increased cocaine abstinence [41]. However, these strategies have been found to be challenging to implement [38]. Future research should continue to seek to identify a novel therapeutic intervention model to reduce morbidity and mortality among cocaine injectors in this setting.

A recent systematic review of mortality among PWIDs found no significant differences in the risk of death by type of primary drug injected [8]. The authors noted some factors that reduced their capacity to detect such differences, including a lack of adjustment for poly-substance use, changes in participants’ drug use habits that may not have been accounted for, and the fact that HIV status was often imprecisely measured [8]. Another systematic review of mortality among cocaine users noted limited data on the extent of elevated mortality among cocaine users [4]. They highlighted limitations of previous studies, including the fact that many cohorts were formed in drug treatment facilities with dependent cocaine users who were at increased risk of premature death and that the prevalence of HIV infection between studies was highly variable [4]. Our study addressed some of the limitations of previous studies by accounting for poly-substance use, following up with participants semi-annually to monitor ongoing drug use habits, and testing for HIV infection at each visit. Furthermore, unlike many previous studies, the present study population was not selected from a drug treatment facility or comprised of cross-sectional examinations of deceased individual’s toxicology reports.

There are several limitations in this study. Firstly, the study sample is not a random sample, and therefore generalizability of our findings may be limited. Secondly, much of the data, particularly regarding drug use patterns, were ascertained through self–reporting. Therefore, our data may have been affected by reporting biases including recall bias and socially desirable reporting. However, we note that this type of data has been commonly utilized in observational studies involving PWIDs and found to be valid [42, 43]. Third, drug use patterns directly before or at the time of death are not described due to the method of self-reporting, which inhibits our ability to ascertain data as proximal to the time of death. Fourth, although migration rates out of the province have been shown to be low, mortality rates may be underestimated, as participants who died outside of BC were not included in the provincial registry. We censored individuals with long durations (>12 months) from behavioural measurement to death on the date of last behavioural measurement. This may have resulted in loss of precision regarding our risk factor measurement. Fifth, for many variables, including alcohol use, we did not have measures that considered refined patterns of use or frequency of use and were often dichotomized into daily versus less than daily use. Lastly, when the endpoint was restricted to accidental mortality, daily cocaine injecting did not remain significantly associated with the time to accidental death, despite a trend towards an association. This may reflect that accidental deaths, including accidental poisonings, assault and accidental falls, are not necessarily predicted by specific drug use patterns. Future research should seek to identify predictors of accidental mortality among PWID. Finally, certain patterns of drug use, such as intranasal cocaine use, are uncommon in this sample and were not measured in our study.

## Conclusions

In summary, daily cocaine injecting was the only drug use pattern independently associated with all-cause mortality among a sample of PWIDs in Vancouver. These findings highlight the need to identify novel effective treatments and harm reduction strategies for cocaine injectors.

## Abbreviations

AHR: Adjusted hazard ratio
ACCESS: AIDS Care Cohort to Evaluate Access to Survival Services
CI: Confidence interval
DTES: Downtown Eastside
HCV: Hepatitis C virus
HIV: Human immunodeficiency virus
PWIDs: Persons who inject drugs
VIDUS: Vancouver Injection Drug Users Study.
